# Correction to: Superinfection and the hypnozoite reservoir for *Plasmodium vivax*: a general framework

**DOI:** 10.1007/s00285-026-02404-3

**Published:** 2026-06-23

**Authors:** Somya Mehra, James M. McCaw, Peter G. Taylor

**Affiliations:** 1 School of Mathematics and Statistics, The University of Melbourne, Parkville, Australia; 2 Centre for Epidemiology and Biostatistics, Melbourne School of Population and Global Health, The University of Melbourne, Parkville, Australia; 3 Mahidol Oxford Tropical Medicine Research Unit, Mahidol University, Bangkok, Thailand

**Correction to: Journal of Mathematical Biology (2024) 88:7** 10.1007/s00285-023-02014-3

There is a typographical mistake in Equation (10) of Mehra et al. (2024), pertaining to the transition rate for mosquito-to-human transmission. The constant $P_M$ in the denominator of Equation (10) should be replaced with $P_H$ to read

$$\begin{aligned} q_{ ( \textbf{h}, \textbf{m}), ( \textbf{h} - \mathbf {e_{i, j}} + \mathbf {e_{i + k, j+1}}, \textbf{m})} = \frac{\beta p \nu ^k}{(\nu + 1)^{k+1}} \frac{m_1}{P_H} h_{i, j}, \, \, i \ge 0, j \ge 0. \end{aligned}$$

This typographical mistake carries through to Equation (16), which is missing the constant $v:= \frac{P_M}{P_H}$ in the numerator and should instead read

$$\begin{aligned} \omega _{ (\mathbf {e_{i + k, j+1}} - \mathbf {e_{i, j}}, 0)}(\textbf{H}, \textbf{M}) = \frac{\beta p v \nu ^k}{(\nu + 1)^{k+1}} M_1 H_{i, j}, \, \, i \ge 0, j \ge 0. \end{aligned}$$

The error persists in Appendix A where we verify conditions for the functional law of large numbers to hold; however, the argument holds with appropriate modification of the constant terms. The system of ordinary differential equations constituting the functional law of large numbers is stated correctly in Equations (20) and (21).
